# An analysis of beak shape variation in two ages of domestic turkeys (*Meleagris gallopavo*) using landmark-based geometric morphometrics

**DOI:** 10.1371/journal.pone.0185159

**Published:** 2017-09-21

**Authors:** Hillary A. Dalton, Benjamin J. Wood, Tina M. Widowski, Michele T. Guerin, Stephanie Torrey

**Affiliations:** 1 Department of Animal Biosciences, University of Guelph, Guelph, Ontario, Canada; 2 Campbell Centre for the Study of Animal Welfare, University of Guelph, Guelph, Ontario, Canada; 3 Hybrid Turkeys, Kitchener, Ontario, Canada; 4 Department of Population Medicine, University of Guelph, Guelph, Ontario, Canada; University of Bari, ITALY

## Abstract

The objective of this study was to assess beak shape variation in domestic turkeys (*Meleagris gallopavo*) and determine the effects of age, sex, and beak size on beak shape variation using geometric morphometrics. Dorsal and right lateral images were taken of 2442 turkeys at 6 and 18.5 weeks of age. Landmarks were digitized in tpsDig in three analyses of the dorsal upper mandible, lateral upper mandible, and lateral lower mandible shape of each turkey at both ages. The coordinate data were then subjected to a principal components analysis (PCA), multivariate regression, and a canonical variates analysis (CVA) with a Procrustes ANOVA in MorphoJ. For the dorsal images, three principal components (PCs) showed beak shape variation ranged from long, narrow, and pointed to short, wide, and blunt upper mandibles at both ages (6 weeks: 95.36%, 18.5 weeks: 92.21%). Three PCs showed the lateral upper mandible shape variation ranged from long, wide beaks with long, curved beak tips to short, narrow beaks with short, pointed beak tips at both ages (6 weeks: 94.91%, 18.5 weeks: 94.33%). Three PCs also explained 97.80% (6 weeks) and 97.11% (18.5 weeks) of the lateral lower mandible shape variation ranging from wide and round to narrow and thin lower mandibles with superior/inferior beak tip shifts. Beak size accounted for varying proportions of the beak shape variation (0.96–54.76%; *P* < 0.0001) in the three analyses of each age group. For all the analyses, the CVA showed sexual dimorphism in beak shape (*P* < 0.0001) with female upper mandibles appearing wider and blunter dorsally with long, curved beak tips laterally. Whereas male turkey upper mandibles had a narrow, pointed dorsal appearance and short, pointed beak tips laterally. Future applications of beak shape variability could have a genetic and welfare value by incorporating beak shape variation to select for specific turkey beak phenotypes as an alternative to beak treatment.

## Introduction

A significant proportion of mortalities and culls in domestic turkeys show signs of injurious pecking, which suggests that this damaging behaviour contributes to decreased productivity and economic losses in commercial production. Injurious pecking also represents a serious welfare concern for domestic turkeys [1–3]. The existing research into the development and causation of injurious pecking in turkeys suggests a complex relationship among multiple factors, but there is little literature on environmental and genetic approaches to reduce this damaging behaviour in modern flocks [2,4]. Current management practices to reduce damage from injurious pecking include a combination of environmental tactics, such as lower light intensities [5–6], reduced stocking densities [7–10], and the provision of enrichment [2,11]—along with physical alterations, such as beak treatment and snood removal [12–13].

Infrared laser treatment is currently the most common form of beak trimming used in domestic turkeys and it is typically performed at the hatchery on day-old turkeys [13]. Compared to the more traditional hot blade method, infrared treatment prevents open wounds, reduces operator error, and reduces behavioural changes immediately after beak treatment as infrared treatment allows the beak tip to wear away gradually over several days [14]. Infrared beak treatment is standard practice to reduce injurious pecking damage in commercial turkey flocks. However, even with improvements of the less invasive infrared techniques, an average of 13% of all turkeys in beak treated flocks still show pecking injuries [13]. Public perception of beak treatment as a painful procedure, performed without analgesia and resulting in loss of beak tip sensation, has led to legislative efforts in several European countries towards banning beak treatment [15–16]. With beak treatment potentially being phased out of commercial practice, there is concern within the industry that environmental approaches alone will not prevent pecking damage from increasing in modern turkey flocks. One potential alternative solution is to examine the phenotypic variation in beak shape to explore the possibility of genetic selection to produce morphological results similar to beak treatment.

Traditional analyses of beak shape in poultry have used linear measurements of length, depth, and width to describe variation in beak morphology [17]. However, these measurements are limited because they convey no geometric data on beak shape and the little information provided of beak shape is not independent of beak size [17–19]. Several studies of laying hens and broiler chickens have used measurements of the dorsal and lateral beak profile to describe differences in beak morphology following beak trimming, but this research was limited to discussion of the variation in beak size rather than true shape differences [20–27]. Landmark-based geometric morphometrics has been successfully applied to study morphological differences in beak shape between several closely related bird species resulting from adaptive radiation to different feeding strategies [17,28–31]. This type of geometric morphometrics visualizes subtle features in the shape variation of a morphological structure as the displacement of biologically homologous landmarks [19,32]. Compared to traditional measurements, geometric morphometrics allows for the separation of size and shape variation. Geometric morphometrics also benefits from heightened statistical power and fewer *a priori* assumptions regarding what measurements should be taken [19,33].

The objective of this study was to evaluate the phenotypic variation in turkey beak shape using landmark-based geometric morphometrics, and to determine if age, sex, and beak size had an effect on the beak shape variation in domestic turkeys. To get a comprehensive understanding of turkey beak morphology, we examined the dorsal and lateral shape variation of the upper and lower mandibles of domestic turkeys in three analyses at two ages. Determining the amount of phenotypic variation in beak shape within domestic turkeys in this study will then allow for an investigation of the genetic basis of beak shape variation. If beak shape variation shows a high response to selection, the possibility exists for future breeding to select for domestic turkeys with a reduced capacity to cause pecking damage as an alternative to beak treatment.

## Materials and methods

The experimental protocol in this study was approved by the University of Guelph’s Animal Care Committee (Animal Utilization Protocol #3171) in strict accordance with the recommendations outlined by the University of Guelph Animal Care Policy and the Canadian Council for Animal Care [34].

### Animals and housing

Beak morphology data were collected on male and female male-line Hybrid convertor turkeys (n = 2442) with known pedigree information. These turkeys came from two groups hatched two weeks apart in May—June 2014. At one day of age, turkeys were de-snooded then individually marked with numbered and bar-coded yellow plastic, tab end wing bands (National Band & Tag Company, Newport, KY, USA). The female and male turkeys from each hatch were housed together in a single power-ventilated, close-sided free-run barn and then separated into single sex flocks at 7 weeks of age in individual barns. The turkeys were housed under standard commercial conditions and fed a standard diet of *ad libitum* feed and chlorinated water from shared feeders and drinkers [35].

### Data collection

**Fig 1:**
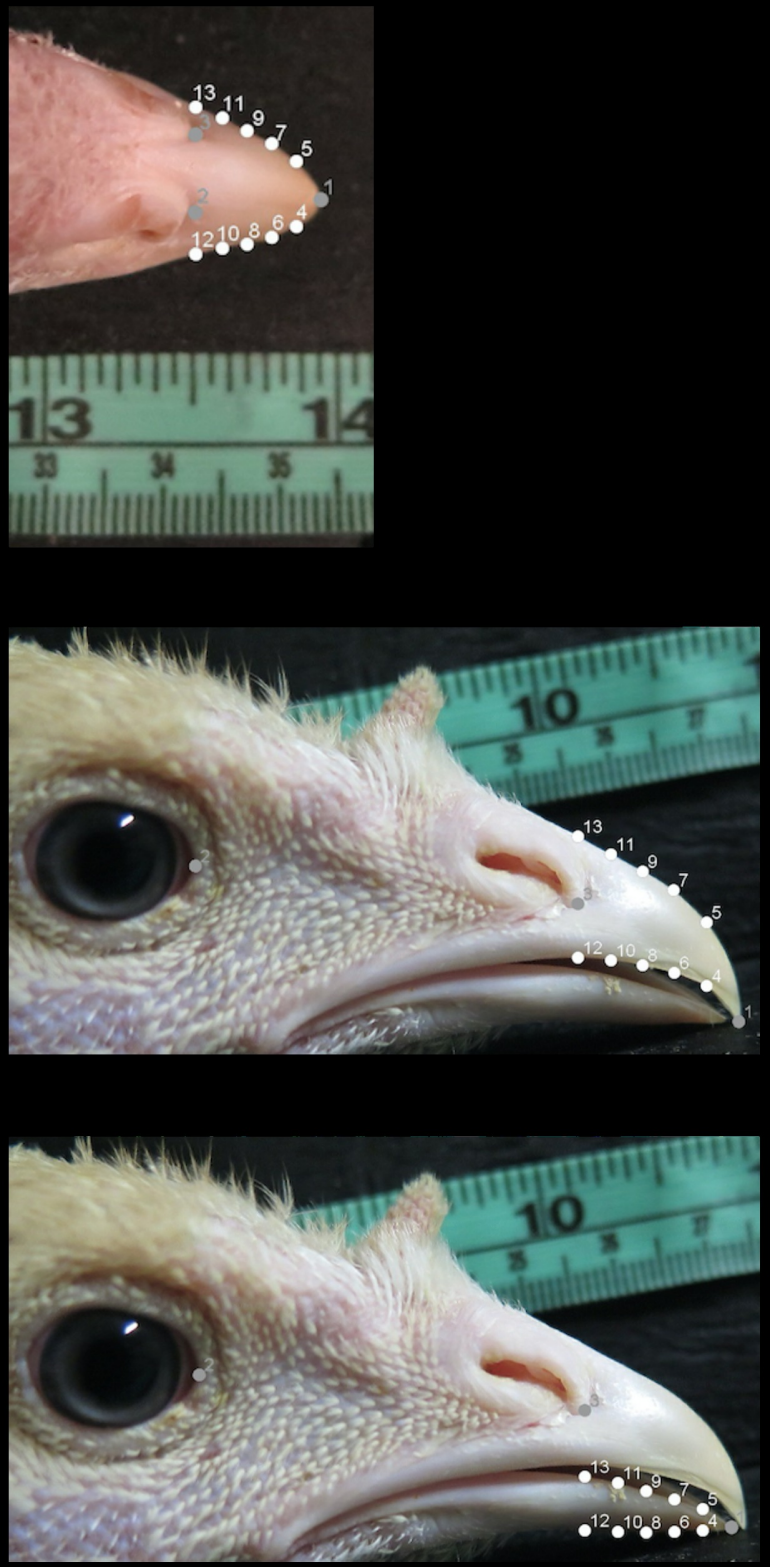
The landmarks and semilandmarks used for the analyses of the dorsal and lateral images. The landmarks (grey) and semilandmarks (white) used for the geometric morphometric analyses of: (A) the dorsal images of the upper mandible (LM 1, beak tip of the upper mandible; LM 2, rostral-most point of the right nostril; LM 3, rostral-most point of the left nostril; LM 4–13, semilandmarks), (B) the right lateral images of the upper mandible (LM 1, beak tip of the upper mandible; LM 2, rostral-most corner of the right eye; LM 3, rostral-most point along the major axis of the right nostril; LM 4–13, semilandmarks), and (C) the right lateral images of the lower mandible (LM 1, beak tip of the lower mandible; LM 2, rostral-most corner of the right eye; LM 3, rostral-most point along the major axis of the right nostril; LM 4–13, semilandmarks) for the domestic turkeys photographed at 6 and 18.5 weeks of age. The semilandmarks included in the three types of analyses were positioned where the beak outline intersected a standardized grid that divided the length of the beak equidistantly.

Each turkey was photographed at two ages: 6 and 18.5 weeks of age. The age class of 18.5 weeks was the average age of turkeys photographed for the second analysis of beak shape between 17–20 weeks of age. Photographs were taken as TIFF image files using a Canon Powershot G16 camera (Canon Canada Inc., Mississauga, ON, Canada) on a black wooden L-shaped platform composed of two black boards (each 20 x 20 cm, length x width) with the horizontal board secured to an adjustable camera tripod (Polaroid Corp., Minnetonka, MN, USA). Both platform boards included a 5 cm ruler for later scaling. A 5 cm plastic strip was also included on the horizontal board to ensure consistent positioning of the turkeys’ heads in the photographs. For each data collection, two photographs were taken from the dorsal and right lateral view of a turkey’s head. Dorsal photographs were taken with the camera at the top edge of the vertical platform board and captured a complete image of a turkey’s head from the beak tip to the base of the skull (Fig 1A). The right lateral images photographed the right side of a turkey’s head from the beak tip to the base of skull (Fig 1B and 1C). Right lateral images were taken with the camera placed on the edge of the horizontal board closest to the photographer. For the dorsal and lateral images, the turkey’s head was positioned along the plastic strip on the horizontal platform board. To photograph a turkey, a technician lifted the turkey underneath the breast and gently held the turkey’s head on the plastic strip of the horizontal platform board while another technician photographed the turkey’s head from both angles.

### Geometric morphometrics

#### Placement of coordinates

Three analyses of beak shape were performed on the dorsal and right lateral images from each turkey at each age. Two analyses of the right lateral image provided a cross section of the beak shape of the upper and lower mandibles separately. A specific set of landmark and semilandmark coordinates (LM) were placed on the beak images in tpsDig version 2.29 [36] in the dorsal, upper mandible, and lower mandible analyses (Fig 1). For the dorsal analysis, three landmarks and 10 semilandmarks were placed along the dorsal outline of the upper mandible (Fig 1A). The right lateral analysis of the upper mandible used three landmarks and 10 semilandmarks on the outer margins of the upper mandible (Fig 1B). Shape analysis of the right lateral view of the lower mandible was also accomplished with three landmarks and 10 semilandmarks outlining the lower beak (Fig 1C) [17,29]. Landmarks are point locations that are biologically homologous between specimens (*e*.*g*., the tip in the upper mandible). In contrast, semilandmarks are points defined by extrinsic criteria and are commonly used to provide more shape information when traditional landmarks are unavailable [19,37]. In this study, semilandmarks were used to capture a complete outline of the beak shape in areas of the beak with no homologous points [17,37].

Before the coordinates were applied, the scaling factor for measurement was set in tpsDig using the ruler that was included in the background of each image. The images were rotated (if necessary) to reduce extraneous variation in the placement of the coordinates. Dorsal images were rotated until a straight line could be drawn from LM 1 and a central line between LM 2 and 3 (Fig 1A). The lateral upper and lower mandible images were rotated to ensure the inferior edge of the lower mandible was straight before proceeding with the placement of coordinates (Fig 1B and 1C). Photographs were excluded from morphometric analysis if the images were blurry or the beaks were damaged. For the lateral lower mandible analysis, photographs were also excluded if the lower mandible was obscured behind the upper mandible. Three technicians were individually responsible for one of the three types of analyses in tpsDig to minimize differences in coordinate placement between images.

The semilandmarks were positioned using a standardized grid and placed where the grid lines intersected the outer margins of the mandible being analyzed [17,31]. For the dorsal images, a straight line was first drawn between LM 2 and 3; then the distance was calculated from this line to LM 1. The upper mandible was then divided into five equal portions along this distance and the semilandmarks positioned along the parallel lines of the grid where it intersected the dorsal outline of the upper mandible (Fig 1A). For the lateral upper and lower mandible images, the distance between LM 3 and the beak tip of the upper or lower mandible (LM 1) was used to divide the beak into five equal sections. The semilandmarks were then placed equidistantly at these grid lines along the right lateral outline of the upper or lower mandible (Fig 1B and 1C) [17,31].

#### MorphoJ shape analysis

Multivariate statistical shape analyses of the turkey beak images were completed using tpsRelw version 1.65 [38] and MorphoJ version 1.06d [39]. Separate MorphoJ analyses were performed for the two age groups and for the dorsal, lateral upper, and lateral lower mandible landmark configurations. After removing the outliers shown by Mahalanobis distance (*i*.*e*., the multidimensional measurement of standard deviation representing the distance between an individual shape measurement and the consensus shape), morphometric beak data was available for 2429 dorsal, 2099 lateral upper mandible, and 2081 lateral lower mandible images for the six-week old turkeys [40]. For the 18.5-week old turkeys, morphometric analysis included 1501 dorsal, 1689 lateral upper mandible, and 1800 lateral images of the lower mandible. For the lower mandible images, LM 3 was excluded from the final MorphoJ analysis because this landmark showed large variation in its placement due to differences in nostril positioning between turkeys with open or closed beaks, which prevented a clear analysis of the shape variation in the lower mandible.

The raw coordinates from each turkey image were first aligned through translation, scaling, and rotation using a generalized least squares Procrustes superimposition algorithm adjusting for sliding semilandmarks in tpsRelw. The aligned shape coordinates were then analyzed in MorphoJ [31–32]. The Procrustes superimposition created a consensus beak shape for the all turkeys within each dataset by identifying the origin point, or centroid, among all the landmarks and semilandmarks in each image, which reduced the dimensionality of the coordinate data from 2*k* to 2*k –* 4 (*k* = total number of landmarks and semilandmarks) [41]. The algorithm then calculated the centroid size for each image as the square root of the sum of squared distances between the centroid and each landmark/semilandmark [32,41–42]. In this study, centroid size served as a measure of each turkey’s beak size independent of its shape [19,41,43]. Therefore, shape was defined as the geometric characteristics of the landmark configuration excluding its orientation, size, and position [41,44].

In MorphoJ, a principal components analysis (PCA) was performed using the covariance matrix of the Procrustes shape coordinates to identify the main orthogonal axes of beak shape variation within each dataset as individual principal components [19]. A canonical variate analysis (CVA) identified morphological shape patterns to distinguish male and female turkeys in each dataset using sex as the group classifier variable. The CVA produced canonical variates to explain the mean beak shape variation differences between the sexes, scaled by the inverse of within-group variation, in the covariance matrix of the Procrustes shape coordinates of each dataset [19,45]. For the principal components and canonical variate analyses, eigenvalues of each component/variate were considered significant for interpretation if they explained ≥5.00% of the total beak shape variation within each dataset. Two multivariate regressions were performed on each dataset to examine the effect of beak size, using centroid size, on the beak shape variation shown in the Procrustes coordinates. The first multivariate regression grouped together the Procrustes coordinates for all the turkeys in a dataset, whereas the second regression pooled the coordinates by sex to determine any sex-specific effects of beak size on beak shape variation [44]. Each multivariate regression included a permutation test of complete independence between the Procrustes shape coordinates and centroid size using 10000 randomization rounds. A Procrustes Anova, using sex as the main effect and wing band number as the individual random effect, then tested the significance of the beak shape differences between male and female turkeys in each dataset [46].

## Results

### Dorsal upper mandible images

**Fig 2:**
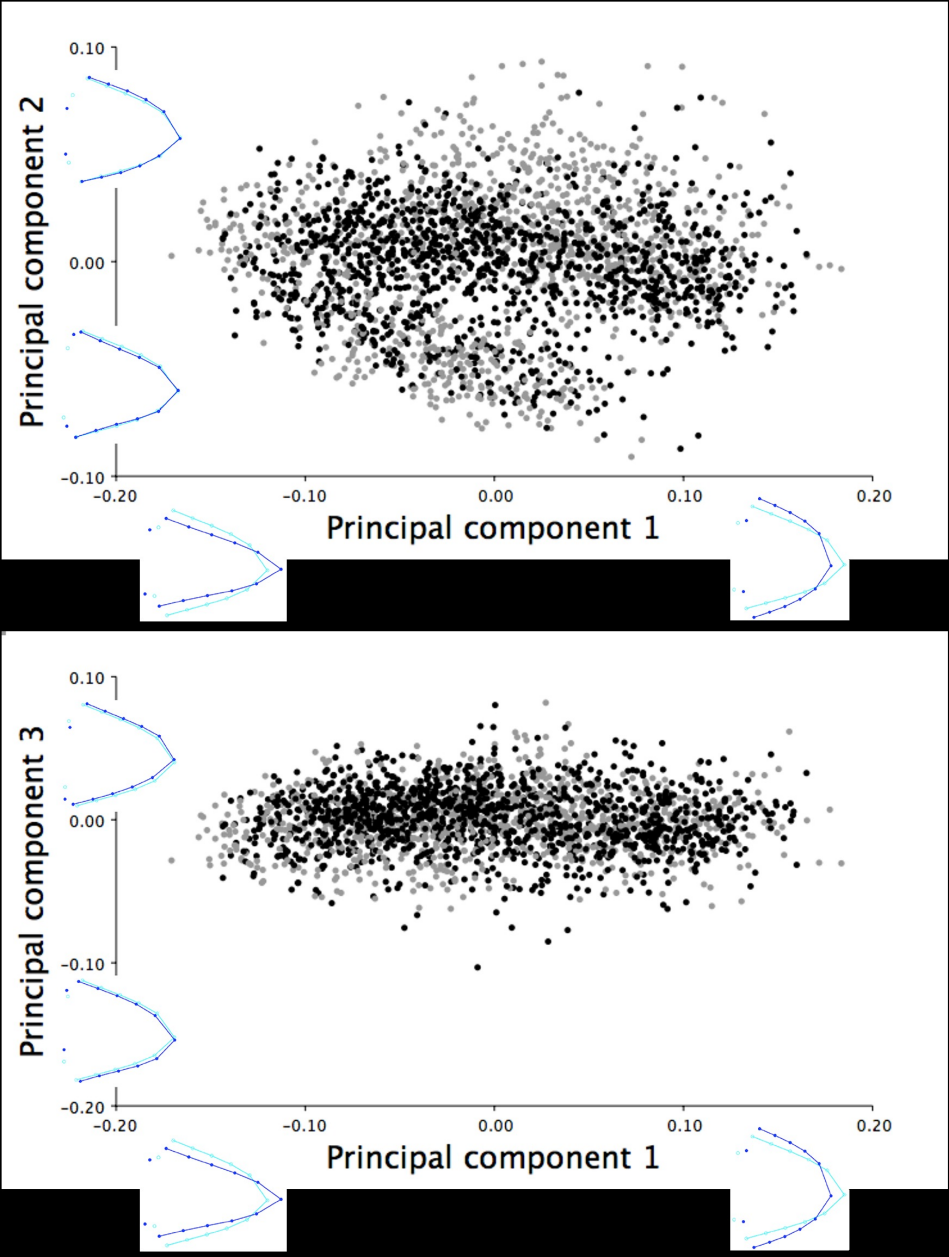
Dorsal shape variation in the upper mandible of the six-week old turkeys. The dorsal shape variation in the upper mandible explained by (A) PC1 and PC2, and (B) PC1 and PC3 for the 2429 male (black) and female (grey) turkeys photographed at six weeks of age. The light blue beak outlines represent the mean dorsal shape of the upper mandible for these six-week old turkeys. The dark blue outlines are visual representations of the dorsal upper mandible shape at the minimum and maximum scores along the axis of each principal component.

The dorsal images of upper mandible included morphometric data from 1186 female and 1243 male turkeys at six weeks of age. The PCA of the dorsal Procrustes shape coordinates for the six-week old turkeys concentrated 95.36% of the explained total variation within the first three principal components (PCs). PC1 explained 74.56% of the shape variation in the dorsal shape profile of the upper mandible ranging between long, narrow beaks with pointed tips to short, wide beaks with blunt tips (Fig 2). PC2 explained 13.93% of the shape variation that showed a shift between a slightly thinner or rounder dorsal outline of the upper mandible and the widening/narrowing of the distance between the nostrils (LM 2 and 3; Fig 2A). In contrast, PC3 accounted for 6.87% of the shape variance that was associated with inferior/superior shifts of the dorsal beak outline (LM 1, 4–13) with an opposite shift in the nostrils (LM 2 and 3; Fig 2B).

**Fig 3:**
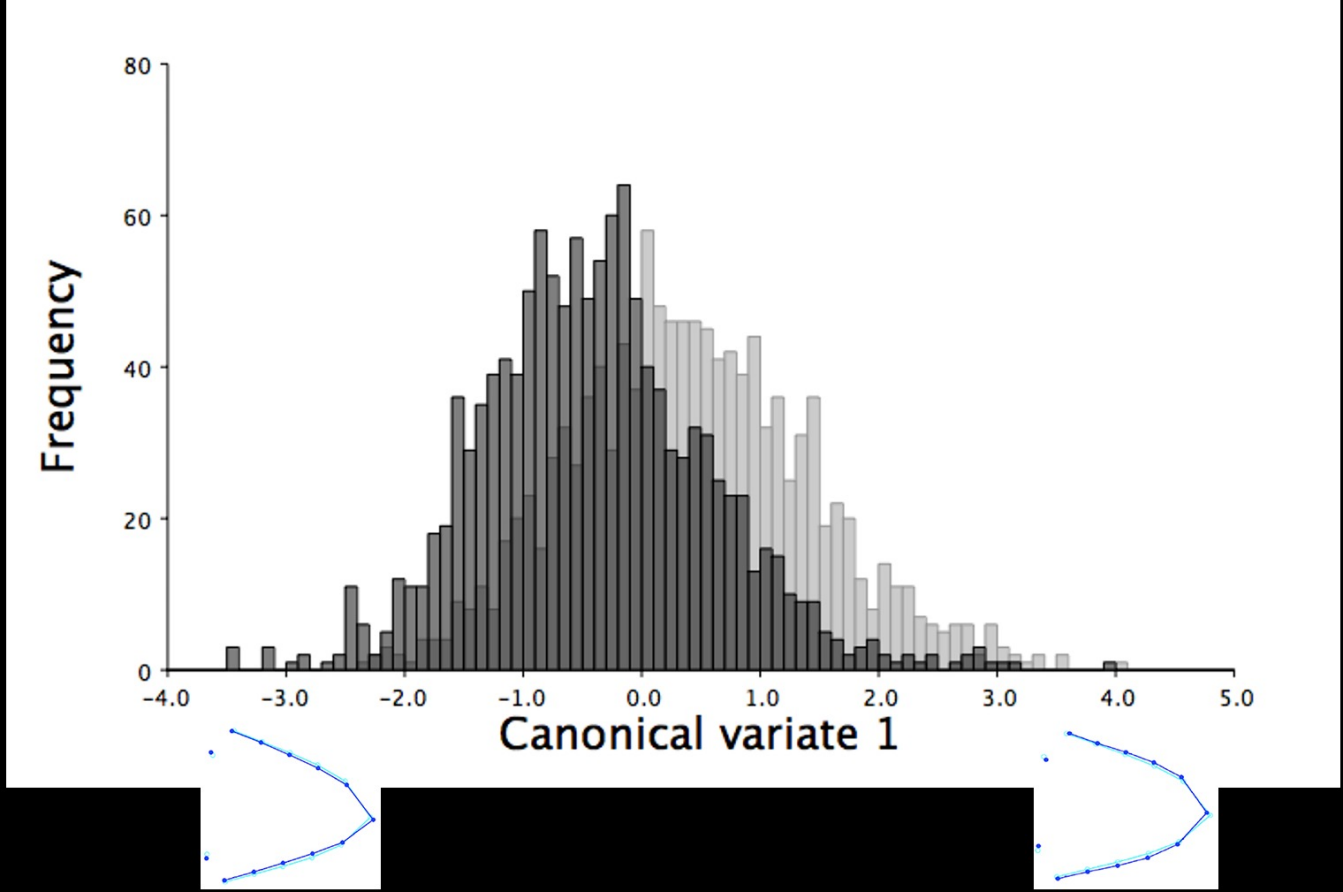
The frequency of six-week old turkeys along the first canonical variate for dorsal shape variation. The first canonical variate accounted for all dorsal shape variation in the upper mandible between male (black) and female (grey) six-week old turkeys. The light blue beak outlines represent the mean dorsal shape of the upper mandible for these six-week old turkeys. The dark blue outlines are visual representations of the dorsal upper mandible shape at the minimum and maximum scores along the axis of the first canonical variate.

Multivariate regression of centroid size showed beak size explained 43.50% of the dorsal shape variation in the upper mandible (*P* < 0.0001), which increased slightly when pooled by sex (45.09%, *P* < 0.0001). Along the axis of centroid size, centroid size explained the variation from long, narrow beaks with pointed tips to short, wide beaks with blunt beak tips for the dorsal upper mandible shape of both male and female turkeys. The CVA produced a single variate that explained 100% of the dorsal beak shape variation between male and female turkeys at 6 weeks of age. This variate showed female turkeys had a slightly wider dorsal upper beak outline shown as an outward shift of the semilandmarks (LM 4–13) and a more blunt beak tip (LM 1) than the pointed beaks of male turkeys at this age (F_22,53394_ = 9.14, *P* < 0.0001; Fig 3).

**Fig 4:**
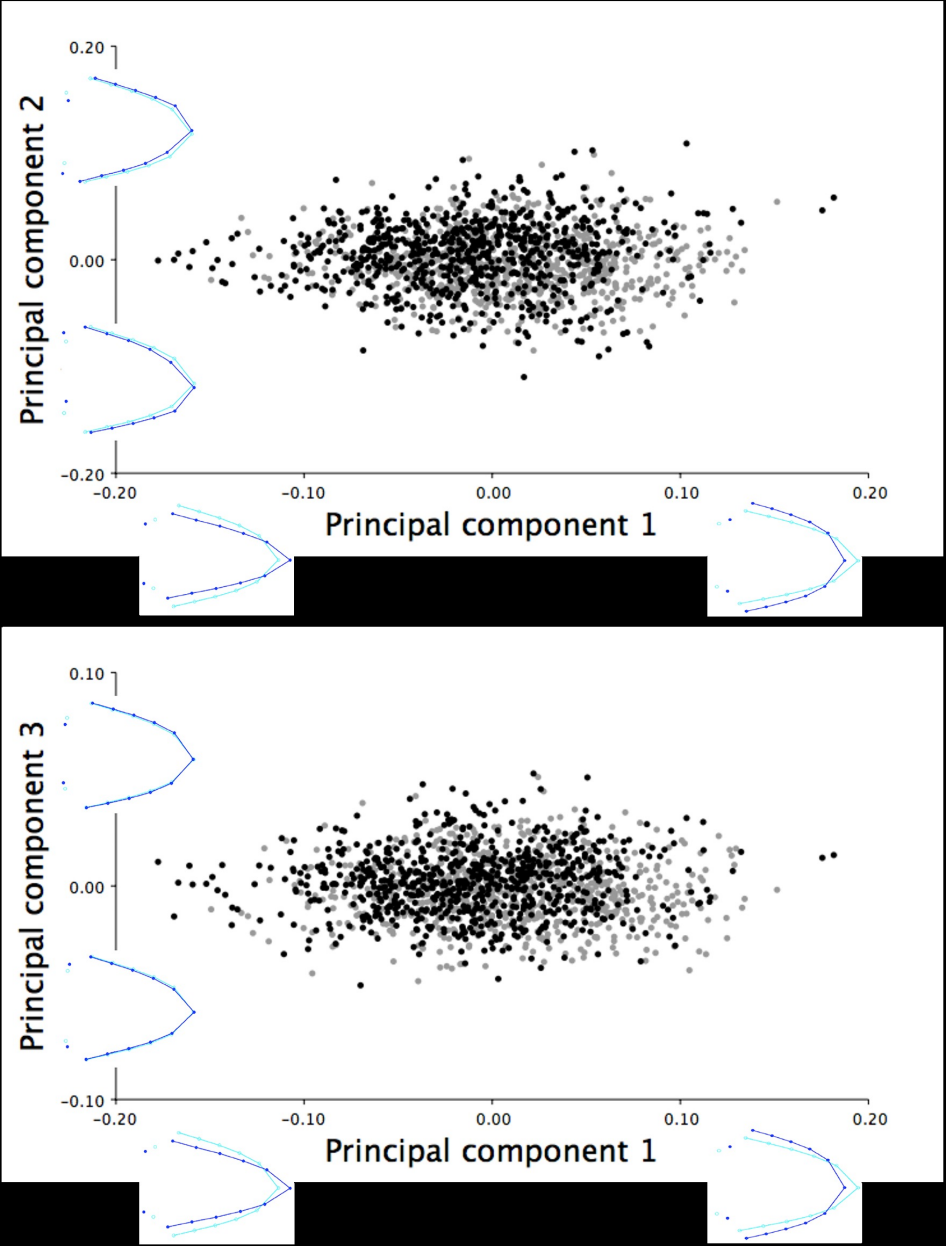
Dorsal shape variation in the upper mandible of the 18.5-week old turkeys. The dorsal shape variation in the upper mandible explained by (A) PC1 and PC2, and (B) PC1 and PC3 for the 1501 male (black) and female (grey) turkeys photographed at 18.5 weeks of age. The light blue beak outlines represent the mean dorsal shape of the upper mandible for these 18.5-week old turkeys. The dark blue outlines are visual representations of the dorsal upper mandible shape at the minimum and maximum scores for this group along the axis of each principal component.

The dorsal upper mandibles images of 773 female and 728 male turkeys at 18.5 weeks of age were analyzed in MorphoJ. Three principal components were extracted from the PCA that explained 92.21% of the total shape variation in the dorsal upper mandible for turkeys at this age. PC1 explained the majority of the shape variation within this group (64.98%) ranging from upper mandibles with a long, narrow dorsal beak shape with pointed tips to short, wide beaks with blunt tips (Fig 4). PC2 referred to 21.61% of explained variation showing the inferior/superior shift of the dorsal beak outline (LM 4–13) with an opposite shift in the positioning of the nostrils (LM 2 and 3; Fig 4A). PC3 (5.62%) explained the narrowing/widening of the dorsal upper mandible outline (LM 4–13) associated with the widening/narrowing of the area between the nostrils (LM 2 and 3; Fig 4B).

**Fig 5:**
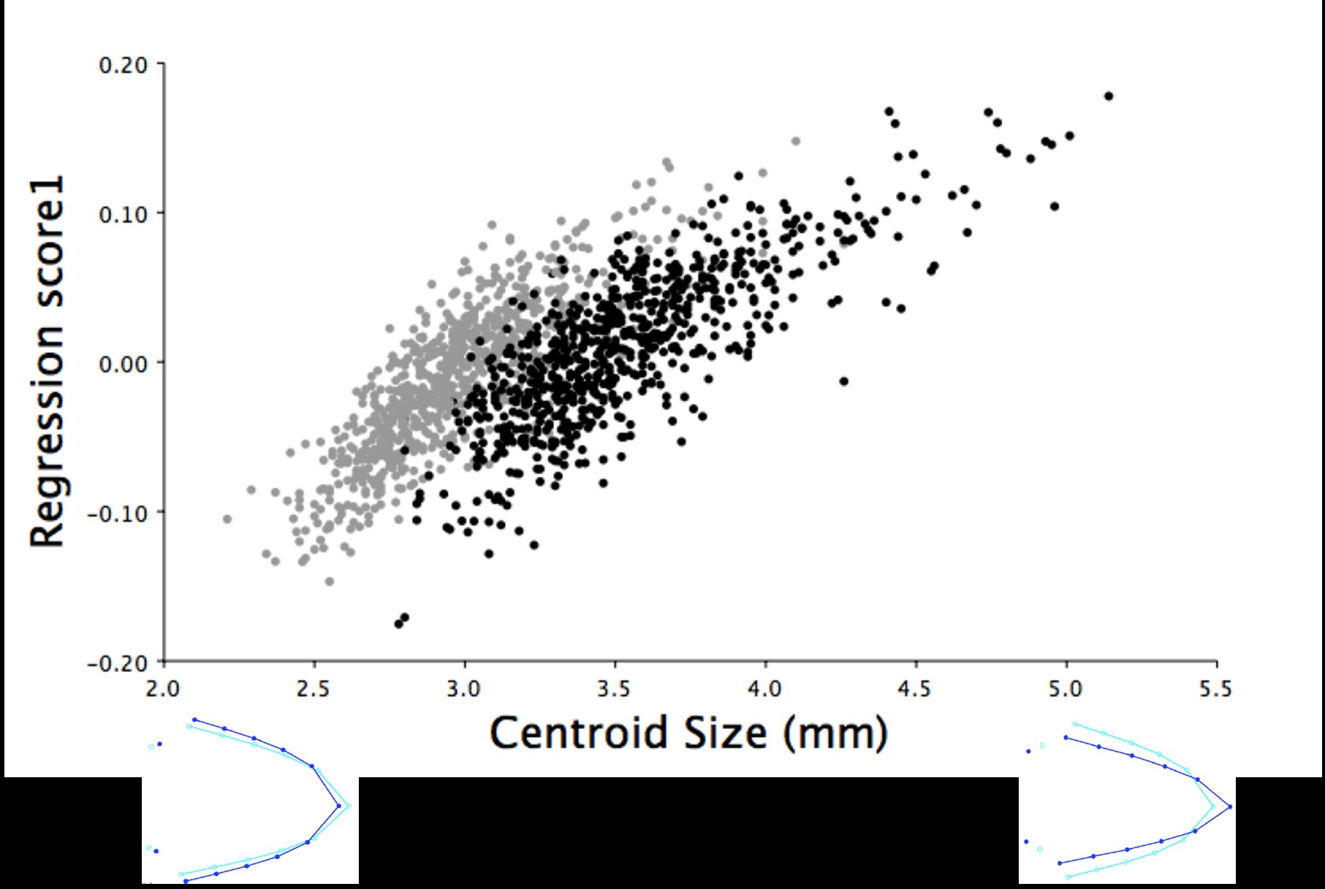
Multivariate regression scores of dorsal shape variation by centroid size for the 18.5-week old turkeys. The multivariate regression scores of the dorsal upper mandible Procrustes shape coordinates by centroid size for male (black) and female (grey) 18.5-week old turkeys (r = 34.54%, *P* < 0.0001). The light blue beak outlines represent the mean dorsal shape of the upper mandible for these 18.5-week old turkeys. The dark blue outlines are visual representations of the dorsal upper mandible shape at the minimum and maximum centroid sizes for this group.

**Fig 6:**
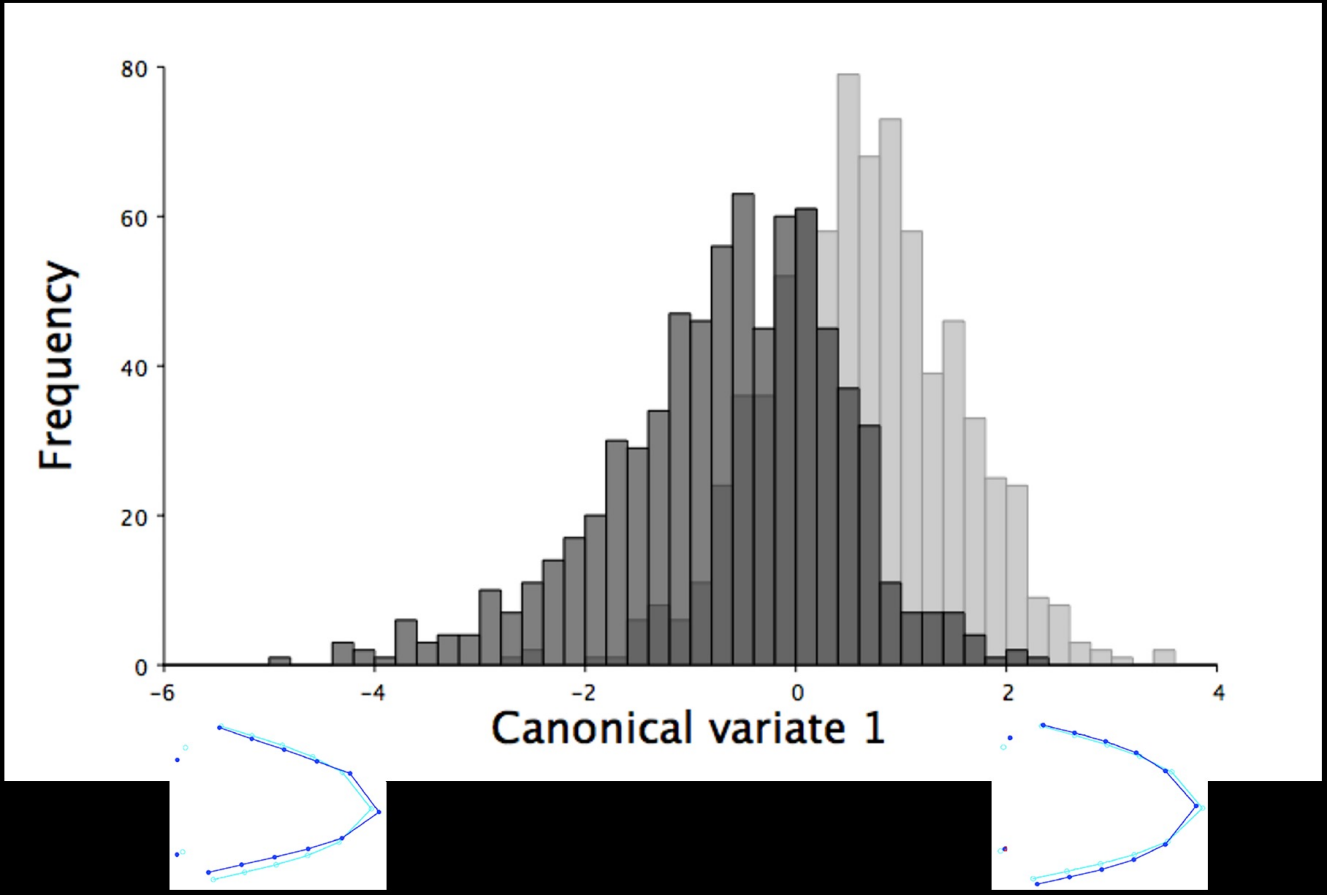
The frequency of 18.5-week old turkeys along the first canonical variate for dorsal shape variation. The first canonical variate accounted for all dorsal shape variation in the upper mandible between male (black) and female (grey) turkeys at 18.5 weeks of age. The light blue beak outlines represent the mean dorsal shape of the upper mandible for these 18.5-week old turkeys. The dark blue outlines are visual representations of the dorsal upper mandible shape at the minimum and maximum scores along the axis of the first canonical variate.

Beak size accounted for 34.54% of the total dorsal shape variation in the upper mandibles for the 18.5-week old turkeys and 41.91% when the data was partitioned by sex (*P* < 0.0001). The larger centroid size of male turkeys (mean centroid size: 3.53 ± 0.01 mm; mean ± standard error of the mean) explained the longer and narrower shape of the dorsal upper mandibles with more pointed beak tips (Fig 5). In contrast, female turkeys at 18.5 weeks of age had smaller centroid sizes (mean centroid size: 2.98 ± 0.01 mm), which explained the shorter and wider shape of the upper mandibles with blunter beak tips (Fig 5). The shape of the upper mandibles also significantly differed between the sexes in the dorsal images of the 18.5-week old turkeys (F_22,32978_ = 51.16, *P* < 0.0001). The CVA of the dorsal upper mandible images produced one variate that explained 100% of the shape variation between the sexes at this age (Fig 6). The dorsal shapes of the upper mandible for females were wider (LM 4–13) with blunter tips (LM 1; Fig 6). In contrast, male turkeys had narrower dorsal upper mandibles (LM 4–13) with more pointed beak tips (LM 1; Fig 6).

### Lateral upper mandible images

**Fig 7:**
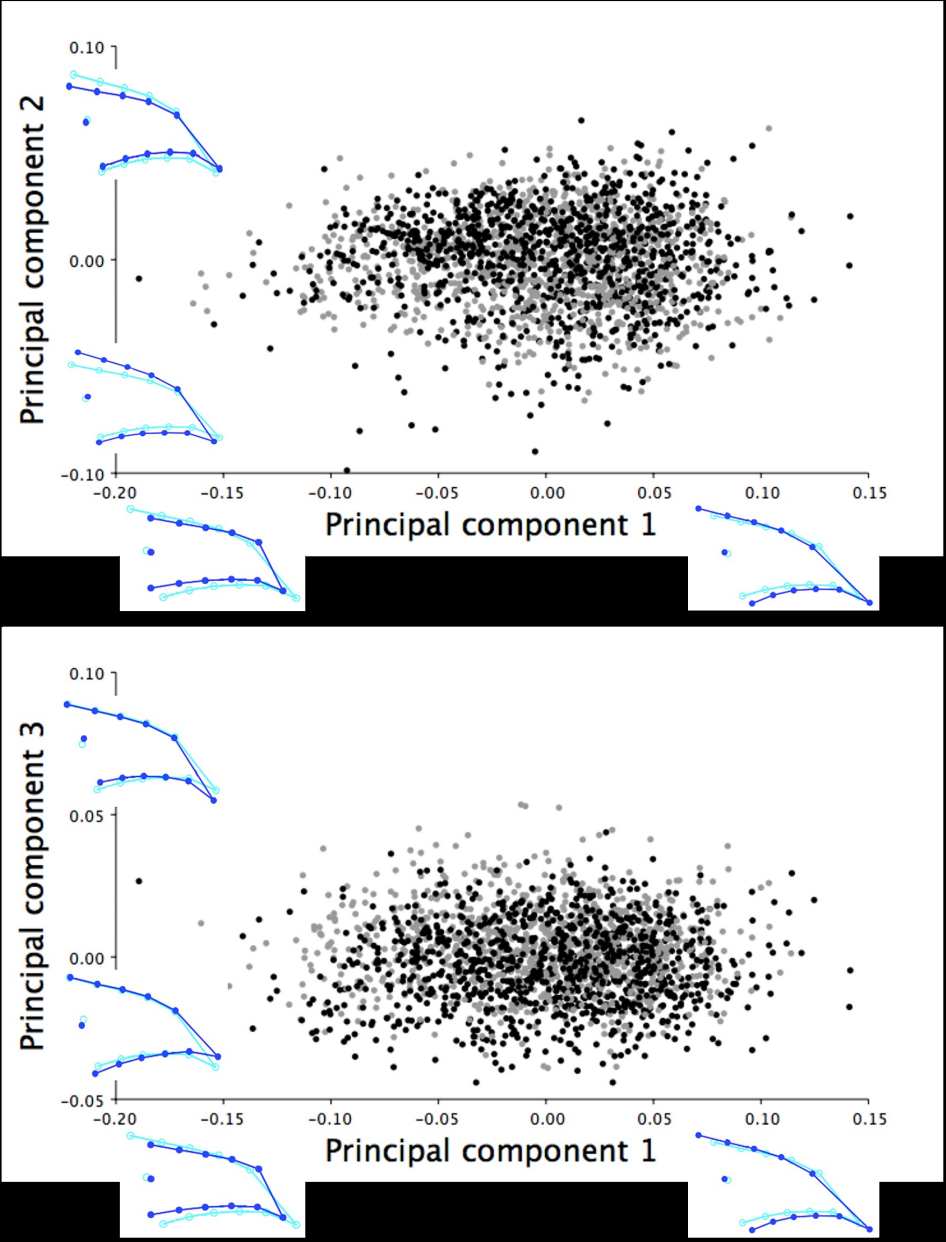
Lateral shape variation in the upper mandible of the six-week old turkeys. The right lateral shape variation in the upper mandible explained by (A) PC1 and PC2, and (B) PC1 and PC3 for the 2099 six-week old male (black) and female (grey) turkeys. The light blue beak outlines represent the mean lateral shape of the upper mandible for these six-week old turkeys. The dark blue outlines are visual representations of the lateral upper mandible shape at the minimum and maximum scores for this group along the axis of each principal component.

For the six-week old turkeys, three principal components were captured from the PCA that cumulatively explained 94.91% of the total variation in right lateral shape of the upper mandible. PC1 accounted for 73.12% of the total shape variation and described the range in the lateral upper mandible shape from short, narrow beaks with short, pointed tips to long, wide beaks with long, curved beak tips (Fig 7). The PC1 shape changes were shown through cranial/rostral shifts in the beak tip (LM 1) and rostral/cranial shifts of the opposite beak margins (LM 4, 6, 8, 10, and 12 *vs*. LM 5, 7, 9, 11, and 13; Fig 7). Both the second (15.01%) and third principal components (6.78%) showed the narrowing/widening of the upper mandible accompanied by the superior and rostral/inferior and cranial shift of the beak tip (Fig 7).

**Fig 8:**
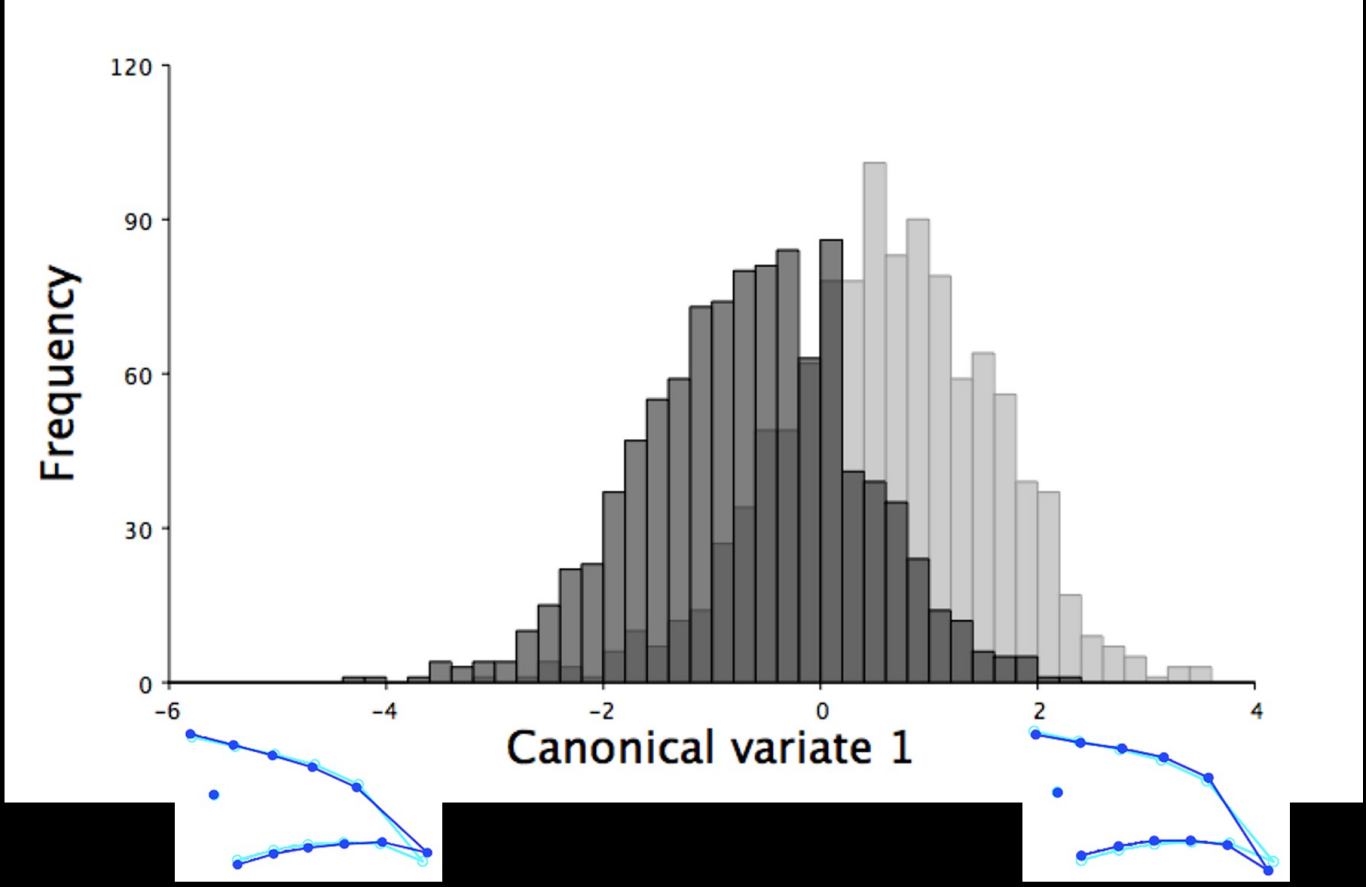
The frequency of six-week old turkeys along the first canonical variate for lateral upper mandible shape variation. The frequency of male (black) and female (grey) turkeys along the axis of the first canonical variate. The first canonical variate accounted for all right lateral shape variation in the upper mandible between male and female turkeys at six weeks of age. The light blue beak outlines represent the mean right lateral shape of the upper mandible for these six-week old turkeys. The dark blue outlines are visual representations of the lateral upper mandible shape at the minimum and maximum scores along the axis of this canonical variate.

Beak size accounted for 13.31% of the total lateral shape variation in the upper mandible for the six-week old turkeys (*P* < 0.0001). Partitioning the data by sex marginally increased the amount of lateral upper mandible shape variation explained by beak size (r = 16.28%, *P* < 0.0001). Along the axis of centroid size, the lateral upper mandible shape of both male and female turkeys varied from wide beaks with more curved tips to narrow beaks with more pointed tips. The CVA produced one variate that explained 100% of the variance in lateral upper mandible shape between the 1089 female and 1010 male turkeys in this group (Fig 8). Fig 8 shows female turkeys at this age had longer, more curved upper mandible tips (LM 1) whereas the upper mandibles of these males had shorter and more pointed beak tips (F_22,46134_ = 15.25, *P* < 0.0001).

**Fig 9:**
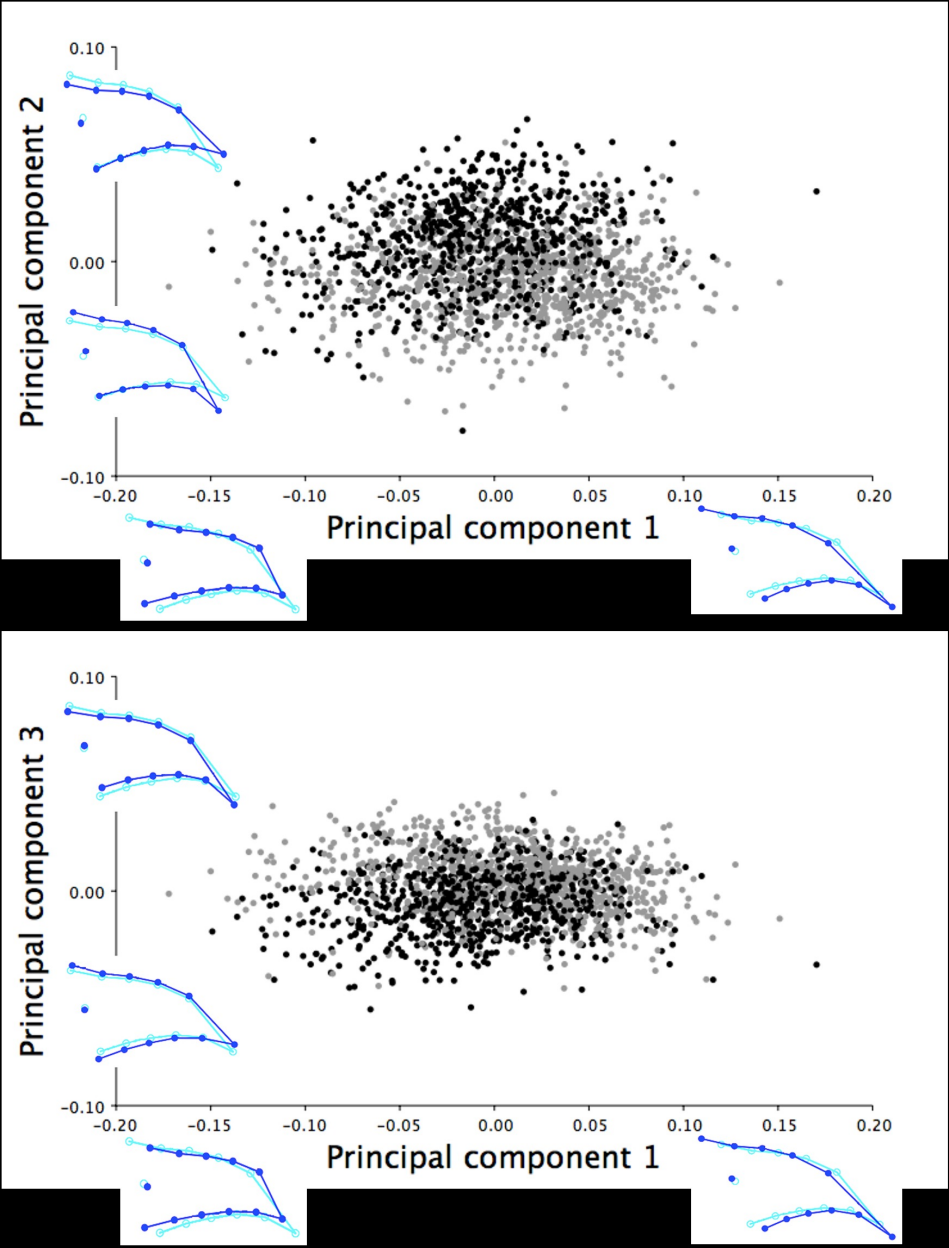
Lateral shape variation in the upper mandible of the 18.5-week old turkeys. The right lateral shape variation in the upper mandible explained by (A) PC1 and PC2, and (B) PC1 and PC3 for 1689 male (black) and female (grey) turkeys photographed at 18.5 weeks of age. The light blue beak outlines represent the mean lateral shape of the upper mandible for these 18.5-week old turkeys. The dark blue outlines are visual representations of the lateral upper mandible shape at the minimum and maximum scores for this group along the axis of each principal component.

For the 18.5-week old turkeys, the lateral images of upper mandible included morphometric data from 932 female and 757 male turkeys. Three principal components were extracted from the PCA, which cumulatively explained 94.33% of the total variation in the right lateral shape of the upper mandible. PC1 explained the majority of the shape variation within this group (72.44%) ranging from short, narrow upper mandibles with short, pointed tips to long, wide beaks with long, curved beak tips (Fig 9). Fig 9 illustrates the PC1 shape changes shown through cranial and superior/rostral and inferior shifts in the beak tip (LM 1) and cranial/rostral shifts of the opposite beak margins (LM 4, 6, 8, 10, and 12 *vs*. LM 5, 7, 9, 11, and 13). The variation in the second (14.28%) and third principal components (7.61%) described the slight widening/narrowing of the lateral upper mandible shape along with the superior/inferior shifting of LM 1, which showed the range from beaks with short, pointed tips to upper mandibles with long, curved beak tips (Fig 9).

**Fig 10:**
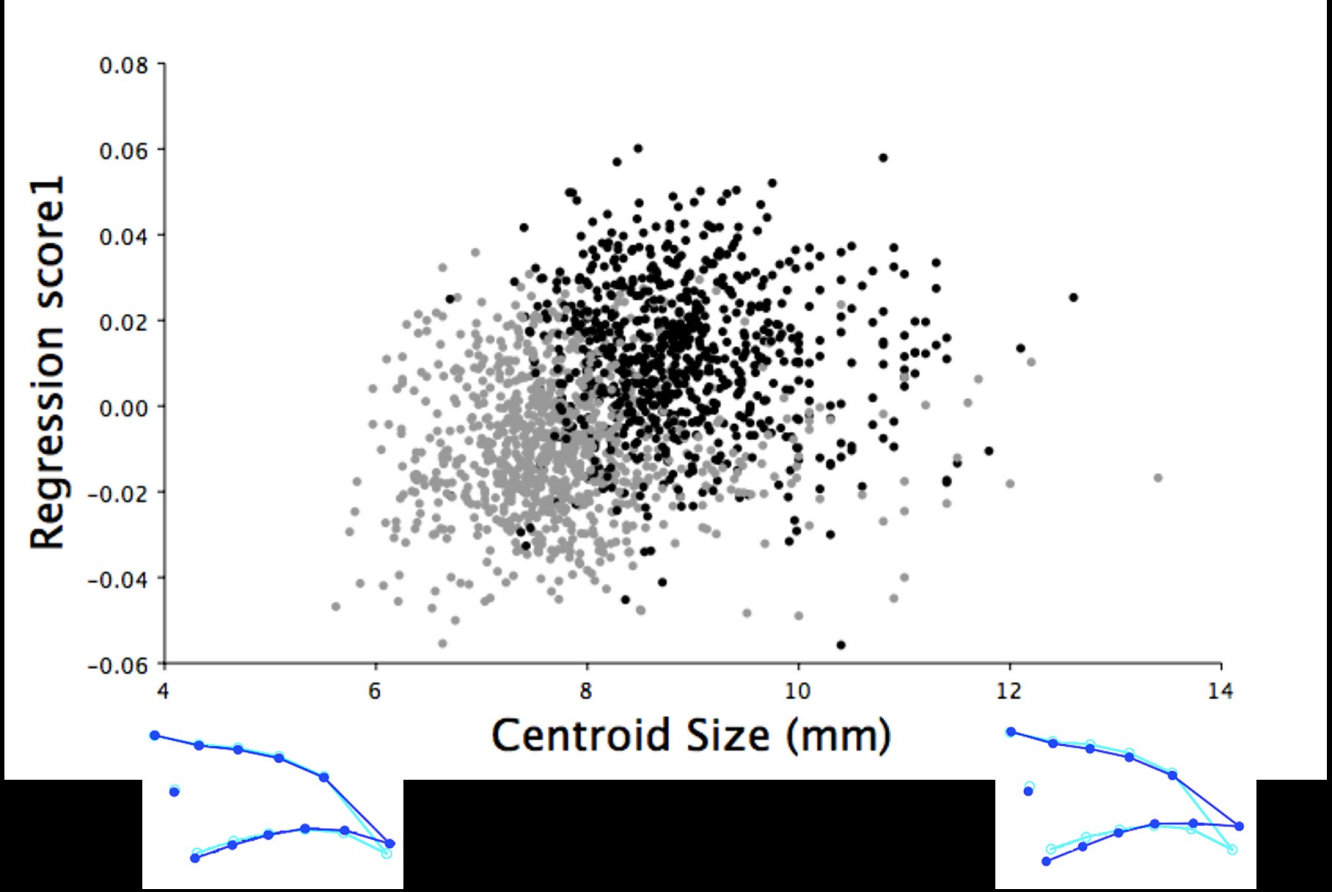
Multivariate regression of lateral upper mandible shape variation by centroid size for the 18.5-week old turkeys. The multivariate regression scores of the right lateral upper mandible Procrustes shape coordinates by centroid size for male (black) and female (grey) 18.5-week old turkeys (r = 0.96%, *P* < 0.0001). The light blue beak outlines show the mean lateral shape of the upper mandible for both male and female turkeys at 18.5 weeks of age. The dark blue outlines are visual representations of the right lateral upper mandible shape at the minimum and maximum centroid sizes for these 18.5-week old turkeys.

**Fig 11:**
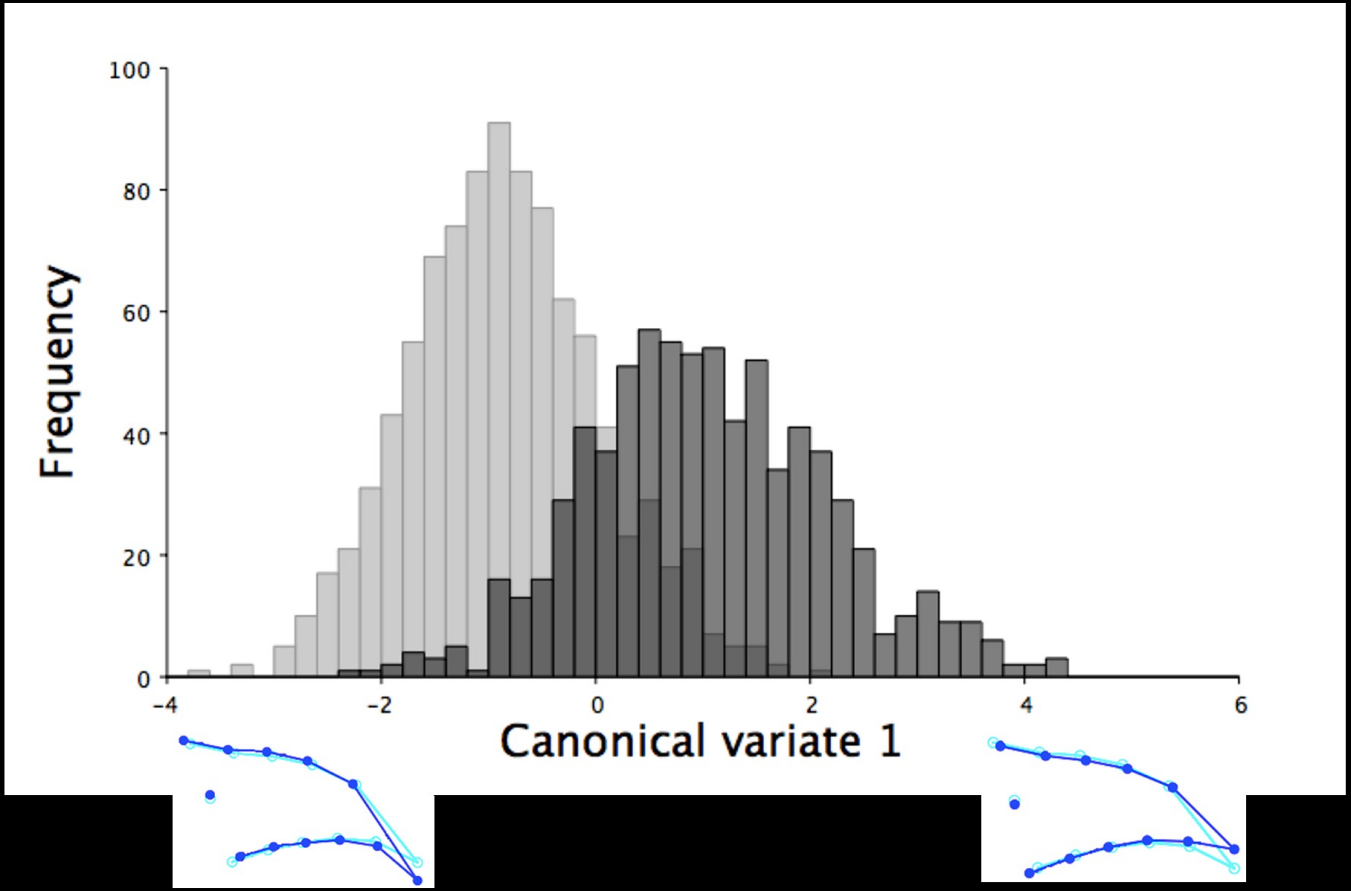
The frequency of 18.5-week old turkeys along the canonical variate for lateral upper mandible shape variation. The frequency of male (black) and female (grey) turkeys along the axis of the first canonical variate. The canonical variate accounted for all right lateral shape variation in the upper mandible between the sexes for the 18.5-week old turkeys. The light blue beak outlines represent the mean right lateral shape of the upper mandible for these 18.5-week old turkeys. The dark blue outlines are visual representations of the lateral upper mandible shape at the minimum and maximum scores along the axis of this canonical variate.

For the 18.5-week old turkeys, beak size only accounted for 0.97% of the total lateral shape variation in the upper mandible, which increased slightly to 1.03% when pooled by sex (*P* < 0.0001). Male turkeys had larger centroid sizes, which explained a more superiorly positioned and pointed upper mandible tip (mean centroid size: 8.92 ± 0.03 mm; Fig 10). Whereas female turkeys at 18.5 weeks of age had smaller centroid sizes (mean centroid size: 7.76 ± 0.03 mm), which explained upper mandibles with more inferiorly positioned and curved beak tips (Fig 10). The CVA yielded one variate that explained 100% of the shape variation in the upper mandible between male and female turkeys at 18.5 weeks of age. This canonical variate showed that female turkeys at 18.5 weeks of age had longer, more curved upper mandible tips whereas males had shorter, more pointed beak tips (Fig 11; F_22,37114_ = 99.28, *P* < 0.0001).

### Lateral lower mandible images

**Fig 12:**
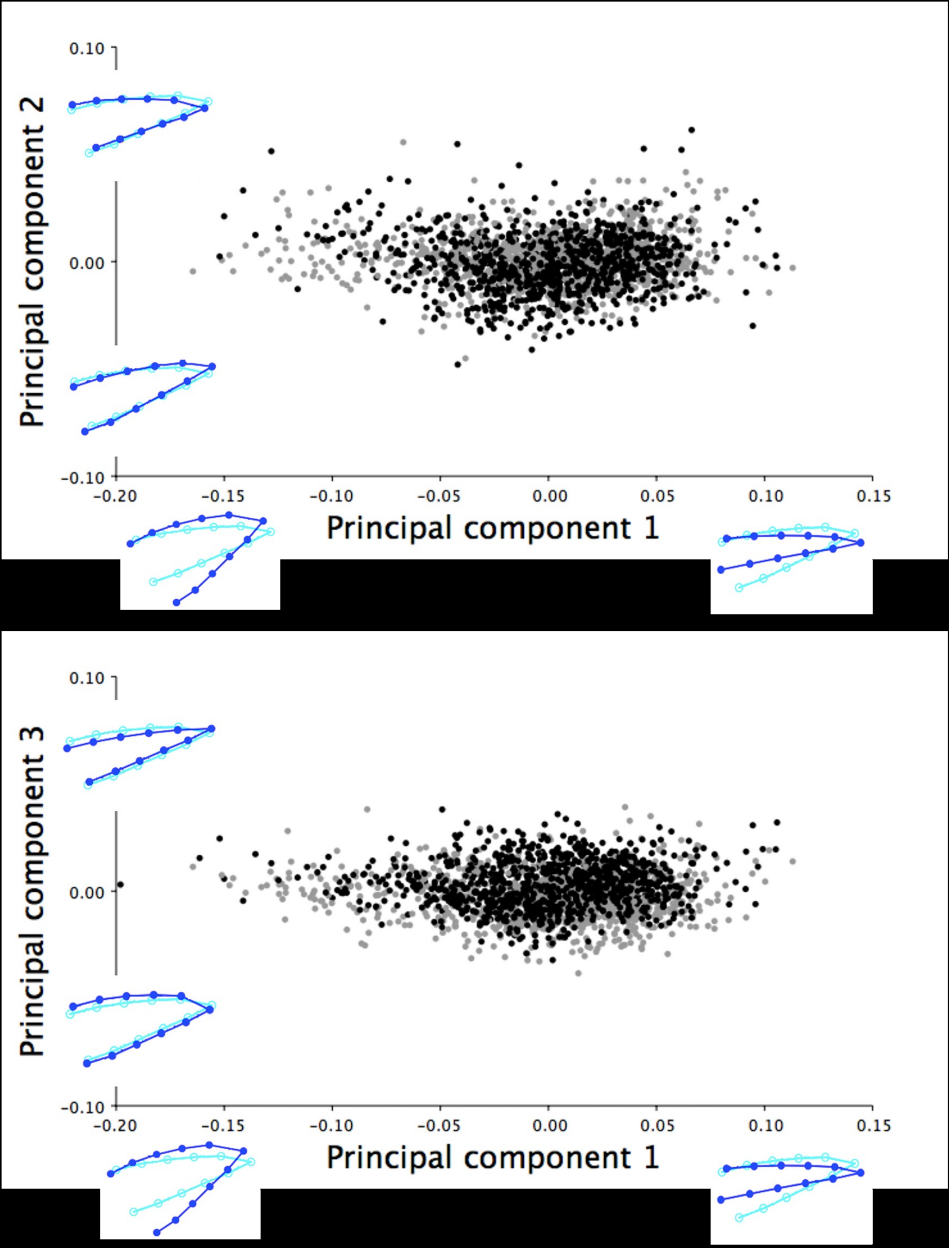
Lateral shape variation in the lower mandible of the six-week old turkeys. The right lateral shape variation in the lower mandible explained by (A) PC1 and PC2, and (B) PC1 and PC3 for 2081 six-week old male (black) and female (grey) turkeys. The light blue beak outlines represent the mean lateral shape of the lower mandible for these six-week old turkeys. The dark blue outlines are visual representations of the lateral lower mandible shape at the minimum and maximum scores for this group along the axis of each principal component.

The PCA of the right lateral lower mandible images produced three principal components that explained 97.80% of the shape variation in the six-week old turkeys. The first principal component accounted for 83.53% of the shape variation, which showed the range from wide and round to narrow and thin lower mandibles (LM 4–13) with an associated superior/inferior shift in the beak tip (LM 1; Fig 12). In contrast, PC2 (7.97%) and PC3 (6.29%) described the widening/narrowing of the lateral shape of the lower mandible (LM 4–13) and the superior/inferior shift of the beak tip (LM 1; Fig 12).

**Fig 13:**
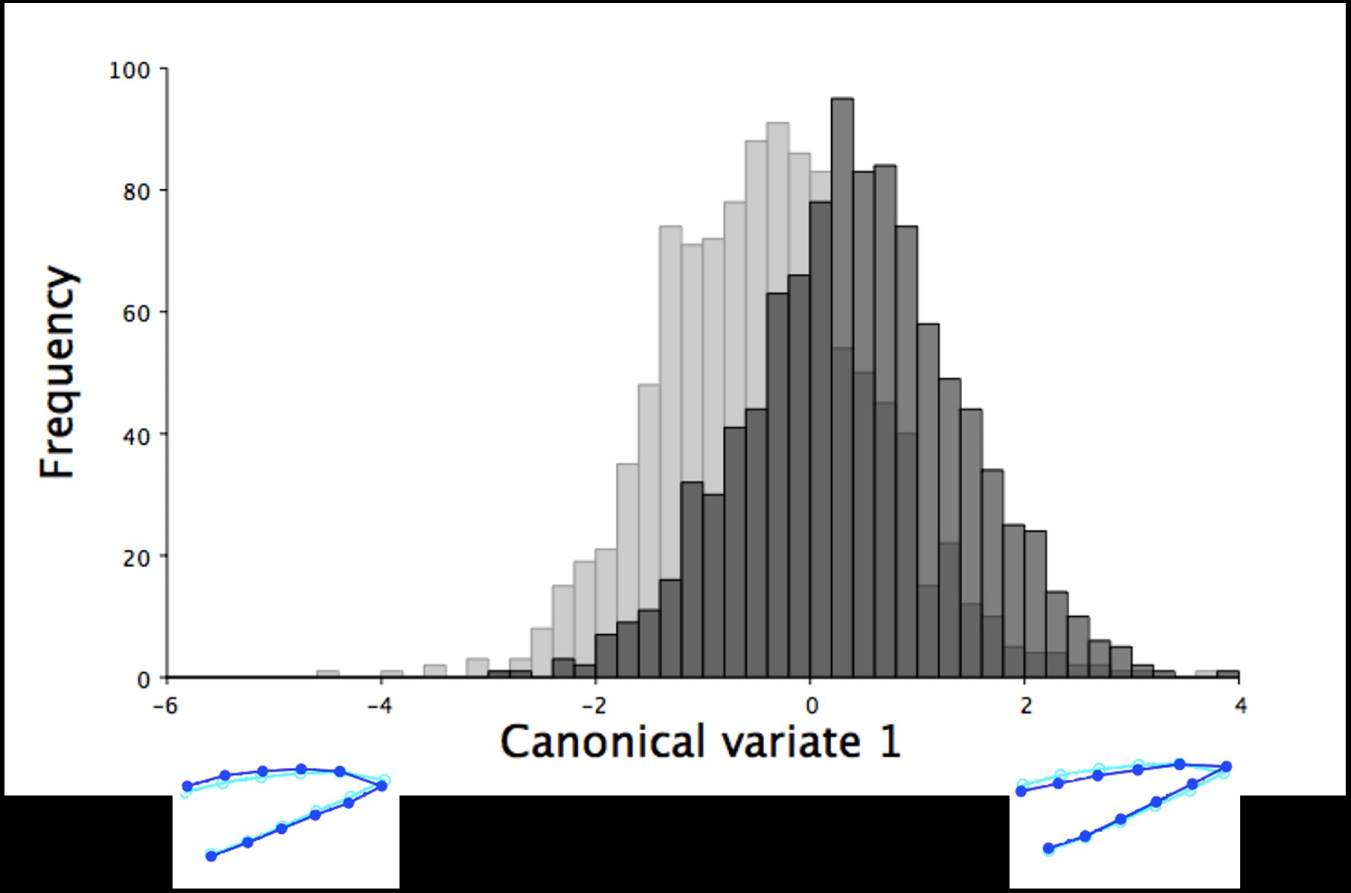
The frequency of six-week old turkeys along the first canonical variate for lateral lower mandible shape variation. The frequency of male (black) and female (grey) turkeys along the axis of the first canonical variate. The first canonical variate accounted for all right lateral shape variation in the upper mandible between male and female turkeys at six weeks of age. The light blue beak outlines represent the mean right lateral shape of the lower mandible for these six-week old turkeys. The dark blue outlines are visual representations of the lateral lower mandible shape at the minimum and maximum scores for this group along the first canonical variate.

Using centroid size, beak size accounted for 34.20% of the total lateral shape variation in the lower mandible for all six-week old turkeys and 54.76% when partitioned by sex into groups of 1068 female and 1013 male turkeys (*P* < 0.0001). Along the axis of centroid size, the lateral lower mandible shape of both sexes varied slightly from narrow to wide beaks. One canonical variate explained 100% of the variation between the lateral lower mandible beak shape of female and male turkeys at six weeks of age. The lower mandibles of female turkeys were rounder and wider with more inferiorly positioned beak tips than the more thin, narrow beaks with superior positioned beak tips of male turkeys at this age (F_20,41580_ = 10.97, *P* < 0.0001; Fig 13).

**Fig 14:**
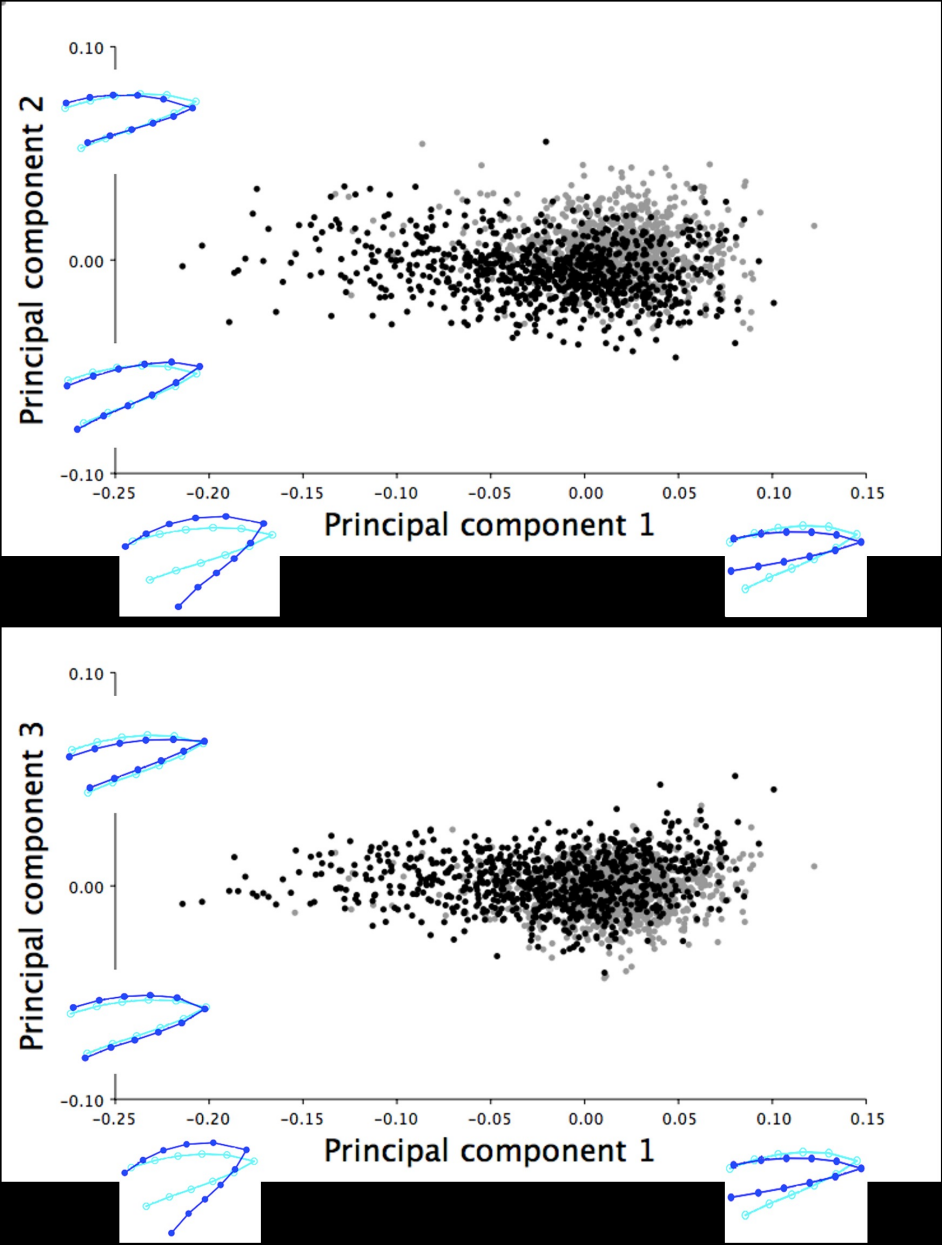
Lateral shape variation in the lower mandible of the 18.5-week old turkeys. The right lateral shape variation in the lower mandible explained by (A) PC1 and PC2, and (B) PC1 and PC3 for 1800 male (black) and female (grey) turkeys at 18.5 weeks of age. The light blue beak outlines represent the mean lateral shape of the lower mandible for these 18.5-week old turkeys. The dark blue outlines are visual representations of the lateral lower mandible shape at the minimum and maximum scores for this group along the axis of each principal component.

The lateral images of lower mandible included morphometric data from 962 female and 838 male turkeys at 18.5 weeks of age. Three principal components from the PCA cumulatively explained 97.11% of the right lateral shape variation in the lower mandible at this age. PC1 (83.14%) explained a majority of the shape variation ranging from wide and round to narrow and thin lower mandibles (LM 4–13) with superior/inferior shifts in the position of the beak tip (LM 1; Fig 14). Similarly, the second (8.33%) and third principal components (5.65%) described smaller changes between the wide and round to narrow and thin lower mandibles (LM 4–13) with inferior/superior shifts of the beak tip (LM 1; Fig 14).

**Fig 15:**
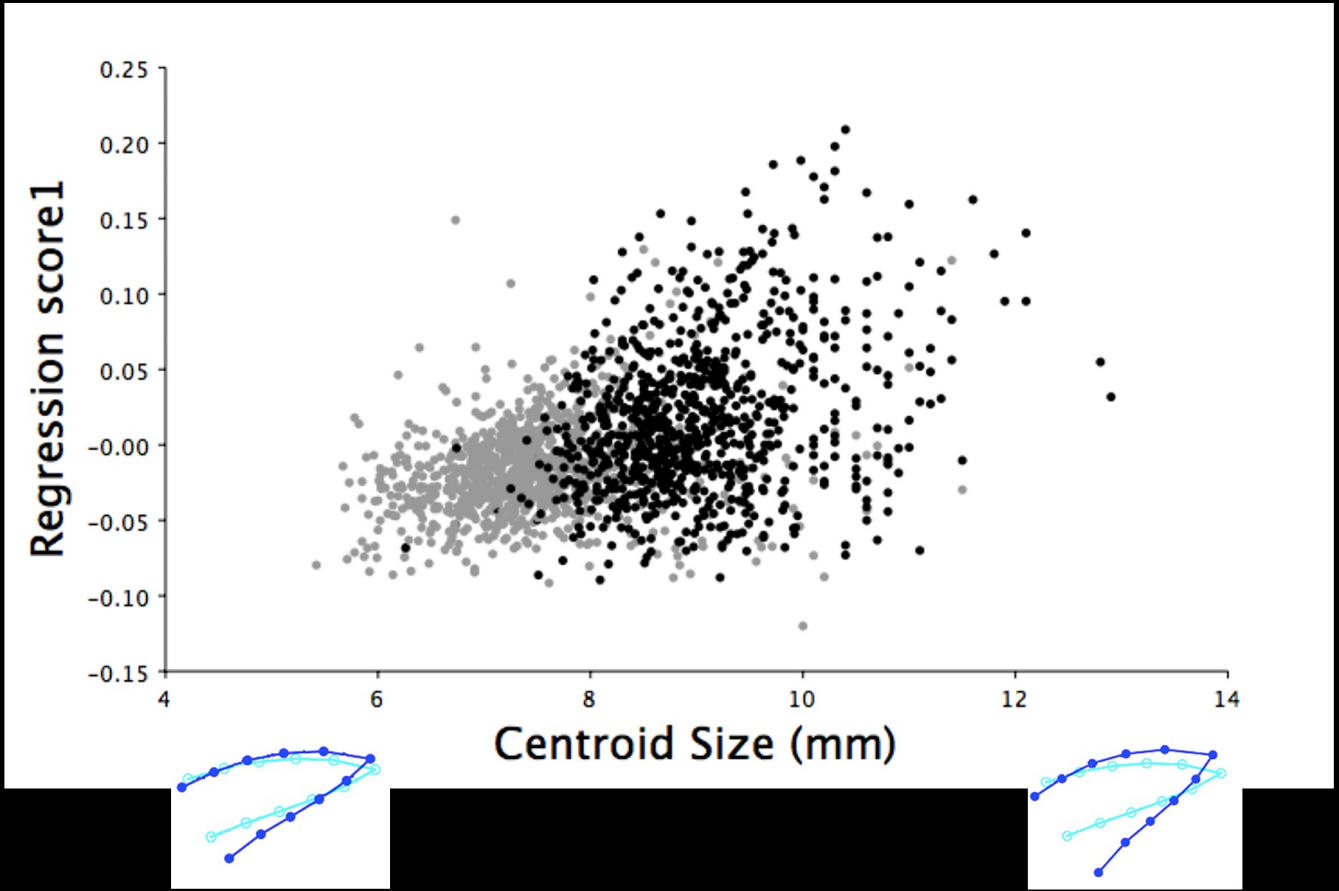
Multivariate regression of lateral lower mandible shape variation by centroid size for the 18.5-week old turkeys. The multivariate regression scores of the right lateral lower mandible Procrustes shape coordinates by centroid size for 838 male (black) and 962 female (grey) 18.5-week old turkeys (r = 15.95%, *P* < 0.0001). The light blue beak outlines show the mean right lateral shape of the lower mandible for both male and female turkeys at 18.5 weeks of age. The dark blue outlines are visual representations of the right lateral lower mandible shape at the minimum and maximum centroid sizes for these 18.5-week old turkeys.

**Fig 16:**
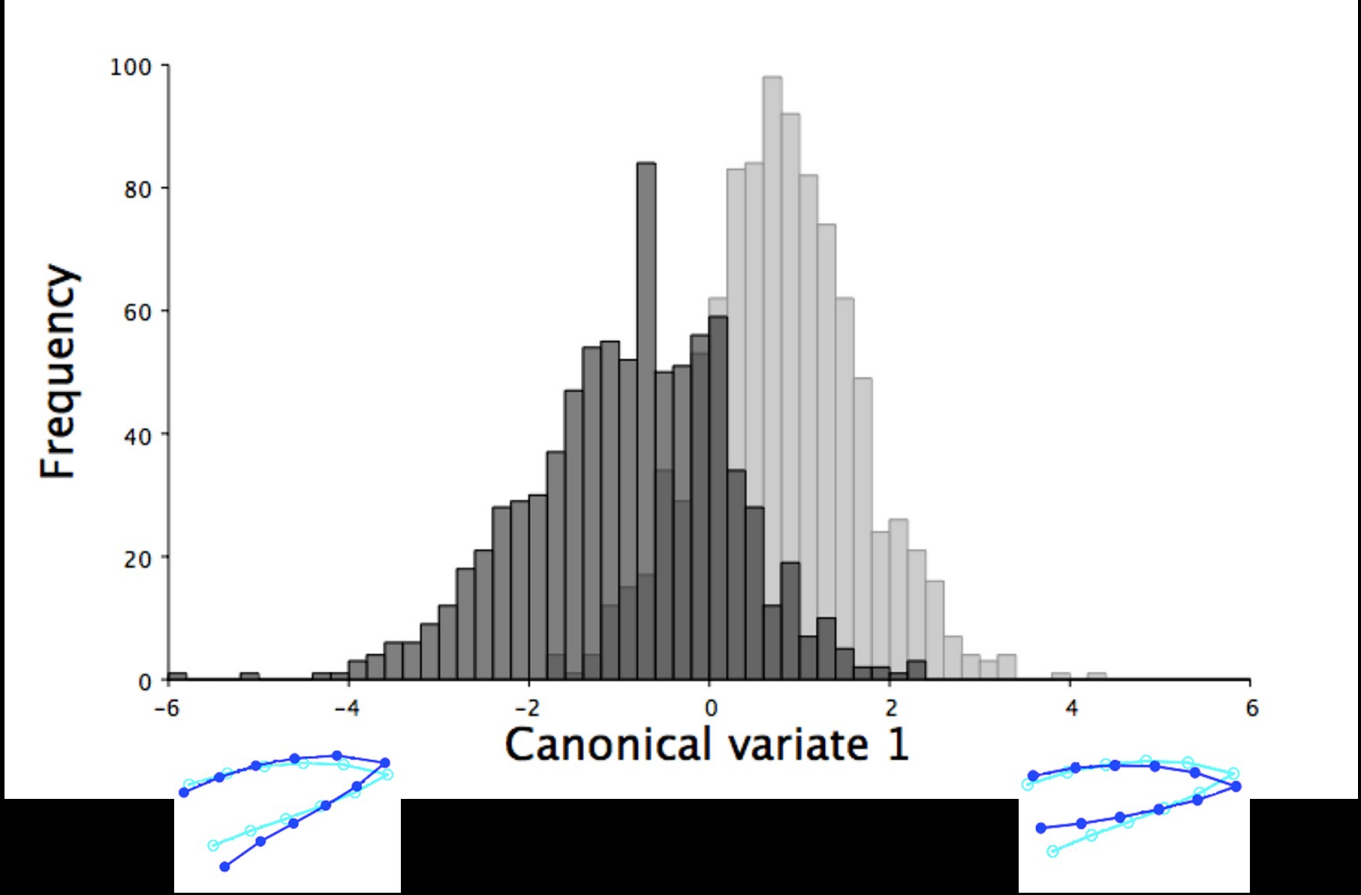
The frequency of 18.5-week old turkeys along the first canonical variate for lateral lower mandible shape variation. The frequency of male (black) and female (grey) turkeys along the axis of the first canonical variate. The first canonical variate accounted for all right lateral shape variation in the lower mandible for male and female turkeys at 18.5 weeks of age. The light blue beak outlines represent the mean lateral shape of the lower mandible for these 18.5-week old turkeys. The dark blue outlines are visual representations of the lateral lower mandible shape at the minimum and maximum scores for these 18.5-week old turkeys along this canonical variate.

At 18.5 weeks of age, beak size accounted for 15.95% of the lateral lower mandible shape variation, which was reduced to 6.29% when grouped by sex (*P* < 0.0001). Male turkeys had larger centroid sizes (mean centroid size: 9.03 ± 0.03 mm) that explained the wider shape of the lateral lower mandibles (LM 4–13) with more superiorly shifted beak tips (LM 1). In contrast, female turkeys at this age had smaller centroid sizes (mean centroid size: 7.44 ± 0.03 mm), which explained more narrow lateral lower mandibles (LM 4–13) with more inferiorly positioned beak tips (LM 1; Fig 15). The CVA of the lateral lower mandible images produced one canonical variate that explained all the shape variation (100%) between male and female turkeys at 18.5 weeks of age (Fig 16). Female turkeys had more narrow, thin lower mandibles (LM 4–13) with an inferiorly positioned tips (LM1) than the more wide, round beaks with superiorly positioned beak tips of male turkeys at 18.5 weeks of age (F_20,35960_ = 245.89, *P* < 0.0001; Fig 16).

## Discussion

Analysis with landmark-based geometric morphometrics showed a wide range of phenotypic shape variation in the beaks of these domestic turkeys. For all three beak analyses, the main axes of beak shape variation were relatively consistent between the two ages. The dorsal outline of upper mandible showed a main axis of shape variation from long, narrow, and pointed to short, wide, and blunt beaks at both 6 and 18.5 weeks of age. Similarly, the majority of shape variation in lateral images of the lower mandible at both ages ranged from wide and round to narrow and thin lower mandibles with superior/inferior shifts in the lower beak tips. The lateral upper mandible showed a main axis of shape variation from long, wide upper beaks with long, curved tips to short, narrow beaks with short, pointed tips at both 6 and 18.5 weeks of age. The main shape axis for these turkeys parallels the main patterns of shape variation (long, narrow, and highly-pointed *vs*. short, wide, and blunt) that have been reported in the lateral upper mandible profiles of other bird species [17,28–31]. These authors proposed that the variation in beak shape across species corresponds most significantly to differences in feeding strategies. For domestic turkeys, the main axis of beak shape variation likely reflects a combination of selection for male-to-male combat and behavioural feeding differences between male and female turkeys.

Sexual dimorphism in beak morphology was apparent between male and female turkeys across both ages and all three analyses. In all three shape analyses, the large degrees of freedom for the Procrustes ANOVA analyses showed a statistical difference in beak shape between the sexes, but also appeared to represent actual biological beak shape differences between the sexes as shown through the phenotypic variation in the CVA figures. The dorsal upper mandible outlines of female turkeys at both ages were significantly wider with blunter tips than males that had narrower beaks with pointed tips. The lateral profile of upper mandible of female turkeys at both 6 and 18.5 weeks of age showed long and curved upper mandible tips, which would appear blunt dorsally. In contrast, the lateral upper mandible shape of male turkeys had short and pointed tips at both ages. The lateral variation in the lower mandible shape for these male and female turkeys showed opposite phenotypes for the two age groups. At six weeks of age, female turkeys had wide, round lower mandibles compared to the narrow, thin lower beak shape for male turkeys. However, the lateral lower mandible shape was narrow and thin for female turkeys and wide and round for male turkeys at 18.5 weeks of age. Sexual dimorphism in beak shape and size is present in other bird species and several hypotheses have been proposed to explain beak morphological differences between the sexes, including divergent feeding strategies, thermoregulation, and sexual selection for male competition and/or female choice [47–49].

In the wild, turkeys reside primarily in same sex groups so the beak shape differences between male and female turkeys might be partially attributed to the specific feed resources that each sex tends to use [47,50–51]. Wild male turkeys use their beaks while fighting to establish dominance or gain access to females in lek-like mating displays, which suggests that the distinct male beak shape phenotype, such as the pointed shape of the upper mandible tips of male turkeys, developed as an effective weaponry for male-to-male conflict [49,52–53]. Research on a lek-breeding species of hummingbird showed that adult males that were more successful in defending displaying territory had more pointed beak tips than subordinate males or females [54]. Wild female turkeys will also select males to breed based on physical qualities of fighting ability, which might include beak size and distinct beak shape characteristics [52–53,55–56]. Sexual selection might also explain the larger beak sizes of males in comparison to female turkeys at 18.5 weeks of age, which also likely corresponds to the larger overall body sizes of males *vs*. female turkeys following sexual maturity. This distinction in beak size between the sexes was not seen between male and female turkeys at six weeks of age [49,57–58]. The larger beak size of male turkeys might also have evolved to serve a thermoregulatory role by aiding in heat dissipation during male courtship displays under warmer conditions [48,59–60]. However, domestic turkeys have undergone extensive selection under commercial production for physical and reproductive characteristics. Therefore, it cannot be assumed that the same biological pressures in wild turkey populations can explain distinctions in the male and female beak shape phenotypes in domestic turkeys.

In the three analyses of beak morphology, variation in beak size predicted varying amounts of the beak shape variation in these turkeys. Beak size explained approximately 35–55% of the explained morphological variation in the lower mandibles of the six-week old turkeys and the dorsal upper mandibles of both ages. In contrast, beak size predicted less than 15% of beak shape variation in the 18.5-week old lateral lower mandibles and the lateral upper mandibles for both ages of turkeys. A recent study [30] showed size-related changes in the beak and braincase accounted for 50% of the lateral upper mandible shape variation between raptor species. For domestic turkeys, specific beak features, such as the dorsal beak shape, might be closely controlled by size, while the shape variation of other beak elements (*e*.*g*., the lateral upper mandible beak tip) might be less constrained by beak size differences. However, more research is needed to substantiate the hypothetical relationships between the size and shape variation in the different beak structures of domestic turkeys.

In summary, landmark-based geometric morphometrics showed a range of phenotypic variation in the shape of dorsal upper mandibles, lateral lower mandibles, and lateral upper mandibles for domestic turkeys at 6 and 18.5 weeks of age. The main axes of shape variation were similar for the two ages, but the beak shape phenotypes of female and male turkeys differed significantly. The role of beak size in predicting beak shape for these turkeys differed between the three analyses and the two age groups. Given the wide phenotypic variation seen in turkey beak shape within this study, the beak shape variables could potentially be used to perform a quantitative genetic analysis to determine the heritability of beak shape variation.

However, the implications from this turkey beak shape analysis is partially limited from only examining male-line turkeys and the reduced sample size for the shape analysis at 18.5 weeks of age. It is unclear if selection pressure for male-line traits, such as larger body weights and fast growth, could have influenced beak shape variation in comparison to female-line turkeys, which are selected for improved fertility, egg production, and egg hatchability [61]. To fully capture the future potential to breed for specific beak features in turkeys, further research should first evaluate if the patterns of beak shape variation are similar within different lines of domestic turkeys. Additionally, the reduction in the sample sizes (13.6–38.2% reduction) from 6 to 18.5 weeks of age, which is attributed to poor photo quality and losses from culling and mortalities, likely impacted the interpretation of beak shape variation for the older group of turkeys.

Subsequent morphometric studies of turkey beak shape variation should analyze the lateral upper and lower mandible shape together to fully understand how the complete shape of the beak varies within domestic turkeys. Before moving forward with selective breeding for beak shape, future research should also examine if distinct beak shape phenotypes influence the feeding behaviour and efficiency of domestic turkeys. Furthermore, there is a need for research to determine the potential capacity for the different beak shape phenotypes to create skin and tissue damage when performing injurious pecking before genetic selection could be considered a realistic alternative to beak treatment in turkeys.

## Supporting information

S1 Dataset The raw x- and y-axis coordinates of the 13 landmarks and semilandmarks from the dorsal images of the 18.5-week old turkeys. (XLSX)

S2 Dataset The raw x- and y-axis coordinates of the 13 landmarks and semilandmarks from the right lower lateral images of the 18.5-week old turkeys. (XLSX)

S3 Dataset The raw x- and y-axis coordinates of the 13 landmarks and semilandmarks from the right upper lateral images of the 18.5-week old turkeys. (XLSX)

S4 Dataset The raw x- and y-axis coordinates of the 13 landmarks and semilandmarks from the dorsal images of the six-week old turkeys. (XLSX)

S5 Dataset The raw x- and y-axis coordinates of the 13 landmarks and semilandmarks from the right lower lateral images of the six-week old turkeys. (XLSX)

S6 Dataset The raw x- and y-axis coordinates of the 13 landmarks and semilandmarks from the right upper lateral images of the six-week old turkeys. (XLSX)
